# Sauchinone Blocks Ethanol Withdrawal-Induced Anxiety but Spares Locomotor Sensitization: Involvement of Nitric Oxide in the Bed Nucleus of the Stria Terminalis

**DOI:** 10.1155/2021/6670212

**Published:** 2021-05-04

**Authors:** Yu Jiao, Sang Chan Kim, Yuhua Wang, Tong Wu, Haifeng Jin, Chul Won Lee, Sook Jahr Park, Bong Hyo Lee, Hee Young Kim, Chae Ha Yang, Zhenglin Zhao, Rongjie Zhao

**Affiliations:** ^1^Department of Psychopharmacology, Qiqihar Medical University, Qiqihar 161006, China; ^2^Medical Research Center, College of Oriental Medicine, Daegu Haany University, Daegu 706-060, Republic of Korea; ^3^Department of Biochemistry, Qiqihar Medical University, Qiqihar 161006, China

## Abstract

Both the positive (manifested by locomotor sensitization) and negative (withdrawal symptoms) reinforcing effects of ethanol (EtOH) involve central nitric oxide (NO) signaling. Sauchinone (a bioactive lignan in *Saururus chinensis*) has been shown to improve methamphetamine-induced behavioral and neurochemical changes via the NO signaling pathway. Thus, this study evaluated the effects of sauchinone on locomotor sensitization and anxiety during EtOH withdrawal (EtOHW). Male adult Sprague-Dawley rats were treated with 1.5 g/kg/day of EtOH (20%, vol/vol) via intraperitoneal injection for 28 days, followed by a 3-day withdrawal. During withdrawal, the rats were given intragastric sauchinone (2.5, 7.5, or 25 mg/kg/day) once a day. EtOH locomotor sensitization was determined by challenging EtOHW rats with 0.75 g/kg EtOH, while EtOHW-induced anxiety was assessed using the elevated plus maze (EPM). None of the three doses of sauchinone affected EtOH locomotor sensitization. However, in the EPM, treatment of EtOHW rats with sauchinone at 7.5 or 25 mg/kg/day increased both the number of entries into and the time spent in the open arms. Moreover, the two doses of sauchinone inhibited the oversecretion of plasma corticosterone during EtOHW. In the bed nucleus of the stria terminalis (BNST), EtOHW increased NO production, enhanced gene and protein expression of both inducible nitric oxide synthase (iNOS) and neuronal NOS (nNOS), and also elevated protein levels of corticotropin-releasing factor, which were all inhibited by 25 mg/kg/day sauchinone. In an in vitro experiment, sauchinone (3, 10, and 30 *μ*M) inhibited H_2_O_2_-stimulated nNOS protein expression in neuronal PC12 cells. Finally, intra-BNST infusion of sodium nitroprusside, a NO donor, after sauchinone (25 mg/kg/day) administration, abolished its expected anxiolytic effect. Taken together, these results indicate that sauchinone attenuates anxiety-like behavior in rats during EtOHW but spares EtOH locomotor sensitization, and the anxiolytic effect is mediated via the NO signaling pathway in the BNST.

## 1. Introduction

Alcoholism imposes a tremendous burden on human health and society, and effective treatments are lacking [1]. Similar to other drugs of abuse, ethanol (EtOH) dependence is elicited and maintained by positive (rewarding effects) and negative (affective withdrawal symptoms) reinforcement mechanisms, which are targeted by pharmacological treatments of alcoholism [2].

Although some rewarding effects of EtOH such as euphoria and gratification in human beings are difficult to be established in animal models, some animal models have nevertheless been used to measure EtOH reward, including rodent models of behavioral and neurochemical sensitization [3, 4]. Locomotor activity is thought to reflect the stimulant-like subjective effects of EtOH, which is putatively regarded as a component of the rewarding effects of drugs of abuse. Increased locomotor activity typically mirrors greater reward intensity [5]. Repeated EtOH exposure enhances locomotor activity in rodents; specifically, EtOH challenge evokes a much greater locomotor response during EtOH withdrawal (EtOHW) [6]. This phenomenon is known as locomotor sensitization and represents a behavioral analogue of the rewarding effect [7]. Moreover, locomotor sensitization is closely associated with challenge-induced sensitization of dopamine release in the nucleus accumbens during EtOHW [4, 7], a form of neurochemical sensitization characterizing the rewarding effect [8]. Thus, rodent locomotor sensitization provides a behavioral and neurochemical readout of the positive reinforcement induced by EtOH and is therefore useful in screening potential pharmacological agents for treating EtOH dependence.

EtOH relapse after periods of abstinence is a major barrier to successful treatment of alcoholism and affective symptoms during EtOHW, such as hyperirritability, anxiety, and dysphoria, constitute the main negative reinforcing factors driving it [9]. EtOHW-induced anxiety, as the chief complaint in alcohol clinics, appears to be the most significant emotional disturbance. Accordingly, self-medication with EtOH to alleviate anxiety remains the main cause for EtOH relapse [10, 11]. In rodents, anxiety-like behaviors in various behavioral tests are exhibited during EtOHW [12] and seem to trigger and facilitate EtOH seeking and self-administration [10, 13]. Undoubtedly, preventing EtOHW-induced anxiety is a promising avenue to restrain EtOH relapse.

Alcoholism is fundamentally a neurocircuitry disorder; neuroadaptation within limbic brain regions involved in addiction forms the biological basis for both behavioral sensitization and withdrawal anxiety [14, 15]. Elevated dopamine in the nucleus accumbens mediates locomotor sensitization [6, 9], whereas increased corticotropin-releasing factor (CRF) signaling in the bed nucleus of the stria terminalis (BNST) underlies EtOHW anxiety [16, 17]. In turn, these neurotransmission changes seem to be maintained by neuromodulators. Nitric oxide (NO), which can serve in this capacity, is involved in both the behavioral sensitization and withdrawal anxiety associated with drugs of abuse. Pretreatment with a nonselective NO synthase (NOS) inhibitor prevents the development and expression of nicotine locomotor sensitization [18], and coadministration of a neuronal NOS (nNOS) inhibitor with EtOH blocks behavioral sensitization [19]. Furthermore, increased NO signaling heightens stress-induced CRF activity in the BNST [20, 21], and the degree of anxiety during morphine withdrawal positively correlates with NOS activity in the hippocampus [22]. Moreover, inhibition of NOS in the dorsal raphe nucleus attenuates EtOHW anxiety [23], and reduced production of NO in the hippocampus appears to mitigate amphetamine withdrawal-induced despair-like behaviors [24].

Herbs from oriental medicine provide a rich pool of bioactive candidates for drug addiction treatment [25]. Evidence from clinical and animal studies indicates that besides the ones typically used to treat neurological disorders, some herbs historically used in nonneurological diseases show potential for treating addiction-related symptoms. For example, the radix of *Glycyrrhiza uralensis*, an herb widely used in the detoxification and treatment of various injuries, has been reported to improve the behavioral and neurochemical disturbances caused by nicotine and methamphetamine dependence [26, 27]. *Saururus chinensis*, similar to *G*. *uralensis* radix, is traditionally used to treat various inflammatory diseases, such as fever, jaundice, and edema [28]. Sauchinone (Sau) is a lignan isolated from *S*. *chinensis*; as with its parent herb, it has anti-inflammatory, antioxidant, and anticancer effects [29, 30]. Lignans belong to a large family of polyphenols that have neuroprotective effects [31]. Previously, Sau was found to inhibit acute methamphetamine-induced hyperlocomotion, as well as the acquisition and expression of methamphetamine-conditioned place preference [32]. It also attenuated repeated methamphetamine-induced neurotoxicity in striatal dopaminergic terminals, which is associated with the negative affect [33, 34]. Crucially, these effects were all linked to the inhibitory effects of Sau on NO production [32, 33, 35]. Since NO signaling plays a critical role in EtOH dependence, these facts give rise to an idea that Sau may have modulatory effects on EtOH dependence.

To test this hypothesis and to widen the pool of bioactive candidates for alcoholism treatment, in this study, the effects of Sau on both EtOH locomotor sensitization and withdrawal anxiety were evaluated in rats. Additionally, the underlying mechanisms were explored, with a focus on NO signaling in the BNST.

## 2. Materials and Methods

### 2.1. Reagents

Sau was isolated from *S*. *chinensis*, and its chemical structure was verified as described previously [30]. Briefly, dried *S*. *chinensis* (Daewon Pharmacy, Daegu, South Korea) was grinded and subjected to methanol extraction (1 : 10, weight/vol) 3 times for 4 hours per time under reflux cooling and distilled under reduced pressure to obtain a methanol extract. The methanol extract was suspended in distilled water and sequentially partitioned with *n*-hexane, trichloromethane, and *n*-butanol. And the *n*-hexane fraction was further fractioned by extensive silica gel chromatography using *n*-hexane: ethyl acetate gradients to obtain Sau. An enzyme-linked immunosorbent assay (ELISA) kit for corticosterone (CORT) was obtained from Abcam (Cambridge, UK), and an assay kit for NO was purchased from Nanjing Jiancheng Bioengineering Institute (Nanjing, China). Primary antibodies against inducible NOS (iNOS), nNOS, CRF, and *β*-actin were supplied by Abcam, and horseradish peroxidase-conjugated secondary antibody was provided by Cell Signaling Technology (Beverly, MA, USA). Sodium nitroprusside (SNP) was purchased from Sigma-Aldrich (St. Louis, MO, USA), and polyethylene glycol (PEG) 400 was obtained from Yakuri Pure Chemical Co. (Kyoto, Japan).

### 2.2. Animals and Experimental Protocols

Male Sprague-Dawley (SD) rats (9 weeks old and weighing 280–300 g) were supplied by the Laboratory Animal Center at Qiqihar Medical University (Qiqihar, China). The rats were housed three per cage with food and water ad libitum. The colony was maintained on a 12 : 12 light/dark cycle with filtered pathogen-free air and kept between 21°C and 23°C with a relative humidity of 50%. Animal experiments were carried out in accordance with the National Institutes of Health Guide for the Care and Use of Laboratory Animals; all procedures were approved by the Animal Care and Use Committee of Qiqihar Medical University (approval number: QMUAECC-2016-16).

Withdrawal from repeated intraperitoneal (i.p.), EtOH (1–3 g/kg/day for 21–28 days) has been previously demonstrated to produce locomotor and neurochemical sensitization, as well as anxiety-like behaviors, in SD rats [4, 36]. Therefore, in this study, to induce locomotor sensitization and anxiety-like behaviors during EtOHW, rats were injected (i.p.) with 1.5 g/kg/day of EtOH (20% vol/vol, dissolved in saline) in their home cages for 28 days, followed by 3 days of withdrawal. During the EtOHW period, the rats received Sau (2.5, 7.5, or 25 mg/kg/day, dissolved in 40% PEG) or vehicle (40% PEG), intragastrically once a day for 3 days in their home cages.

**Figure 1:**
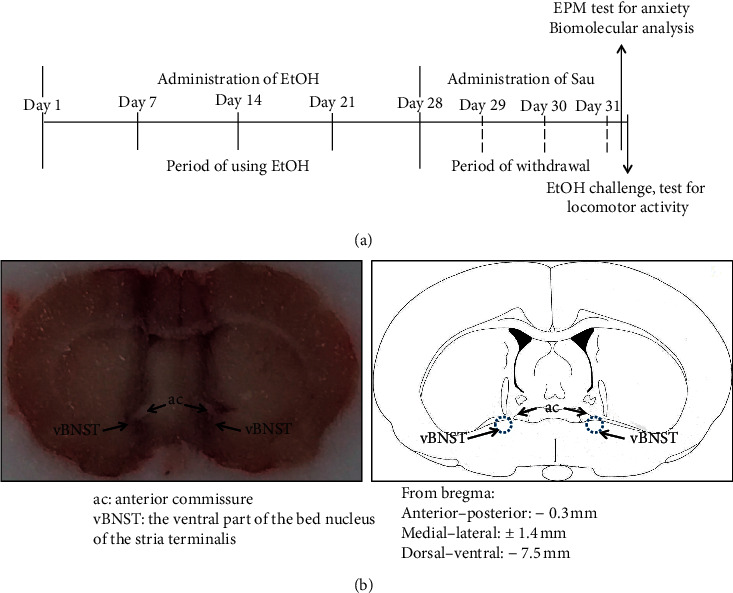
Schedule for EtOH withdrawal anxiety and locomotor sensitization (a) and the representative microphotograph of bilateral microinfusion positions in the BNST (b).

To evaluate EtOH locomotor sensitization, rats were moved to the locomotor testing boxes immediately after the final dose of Sau. Following a 30-min habituation period, the rats were challenged with 0.75 g/kg EtOH and then left in the boxes for an additional 60 minutes, while locomotor activities were measured (Figure 1(a)). The experimental groups were as follows: saline/vehicle/saline (*n* = 8), saline/vehicle/EtOH (*n* = 8), EtOH/vehicle/saline (*n* = 8), EtOH/vehicle/EtOH (*n* = 8), EtOH/Sau2.5/EtOH (*n* = 8), EtOH/Sau7.5/EtOH (*n* = 8), and EtOH/Sau25/EtOH (*n* = 8).

Another cohort of rats were tested in the elevated plus maze (EPM) 30 min after the last intragastric Sau dose to measure their anxiety-like behaviors. The experimental groups were as follows: saline/vehicle (*n* = 8), EtOH/vehicle (*n* = 8), EtOH/Sau2.5 (*n* = 8), EtOH/Sau7.5 (*n* = 8), = EtOH/Sau25 (*n* = 8), and saline/Sau25 (*n* = 8). Immediately following the EPM, the animals were euthanized and decapitated. The brains were removed and stored at −80°C until the BNST was excised (coordinates from bregma [37]: anterior-posterior, −0.3 mm; medial-lateral, ±1.4 mm; and dorsal-ventral, −7.5 mm) for subsequent ELISA, quantitative polymerase chain reaction (qPCR), and Western blot analyses. Additionally, trunk blood was collected to evaluate plasma CORT concentrations (Figure 1(a)).

### 2.3. Locomotor Activity Test

Locomotor activity was measured in a rectangular box (60 × 60 × 50 cm^3^) with floors and walls made of clear acrylic panels painted black. The box has a video camera located above the center of the floor. Rats' locomotor activity was recorded and analyzed with a video tracking system (Shanghai Xinruan Technology Co., Shanghai, China).

### 2.4. The EPM

Anxiety-like behaviors in rats were evaluated in the EPM [12]. Briefly, the EPM is cross-shaped and consists of two opposing open arms (50 cm long × 10 cm wide) with no walls and two opposing closed arms with dark acrylic walls (40 cm high). The arms are raised 50 cm from the floor and monitored with a video-tracking system (Shanghai Xinruan Technology Co.). The testing room was maintained under indirect dim light (2 × 25 W) to encourage rats to explore the arms. At the start of the test, each rat was placed in the center of the maze, and the number of arm entries and time spent in each arm by the rat were monitored for 5 min. The percentages of the entries made and time spent in the open arms were calculated as follows:

(1) $$\begin{array}{c} \text{\% }{\text{entry}}_{\text{open arms}}=\frac{{\text{entry}}_{\text{open arms}}}{{\text{entry}}_{\text{open arms}}{+\text{entry}}_{\text{closed arms}}}\times 100\text{,}\\ \text{\% }{\text{time}}_{\text{open arms}}=\frac{{\text{time}}_{\text{open arms}}}{{\text{time}}_{\text{open arms}}{+\text{time}}_{\text{closed arms}}}\times 100\text{.} \end{array}$$

### 2.5. Cell Culture and Treatment

A differentiated PC12 cell line (derived from rat pheochromocytoma cells) was provided by the American Type Culture Collection (Rockville, MD, USA). The PC12 cells were cultured in Dulbecco's modified Eagle's medium (Invitrogen, Carlsbad, CA, USA) containing 10% fetal bovine serum, 50 units/mL penicillin, and 50 mg/mL streptomycin that was maintained at 37°C in a humidified 5% CO_2_ atmosphere. After the cells reached a confluence of approximately 80%, they were subcultured. To determine the effects of Sau on oxidative stress-induced nNOS expression, the subcultured PC12 cells were pretreated with 3, 10, or 30 *μ*M Sau dissolved in dimethylsulfoxide (DMSO); after 60 min, they were exposed to 75 *μ*M H_2_O_2_ for 24 hours. The cells were then harvested for further biochemical assays.

### 2.6. Cell Viability Assay

PC12 cells were cultured in 24-well plates (density of 5 × 10^4^ cells per well). The cells were stained with 0.25 mg/mL MTT for 2 hours after being treated with 75 *μ*M H_2_O_2_, Sau, or their combination. The media were removed from the wells, and the formazan crystals were dissolved by adding DMSO. The absorbances were read with a microplate reader (Tecan Infinite M200; Tecan, Mannedorf, Switzerland) at 570 nm. The relative cell viability was quantified by the following formula:

(2) $$\begin{array}{c} \text{\% cell viability}=\frac{\left(\text{absorbance of treated sample}\right)}{\left(\text{absorbance of control}\right)}\times 100\text{.} \end{array}$$

### 2.7. Measurement of Plasma CORT Concentrations and NO Levels in the BNST

To separate the plasma, trunk blood (1 mL) was mixed with 20 *μ*L ethylenediaminetetraacetic acid (20 mg/mL) in a chilled tube and centrifuged at 1,500 ×g for 10 min at 4°C. The BNST tissues were excised from the stored rat brains, homogenized in ice-cold saline (pH = 7.4), and centrifuged at 2,500 ×g for 15 min at 4°C. The supernatants were collected. Plasma CORT concentrations were determined with the ELISA kit (Abcam) and expressed as ng/mL, while NO levels of the supernatants were evaluated via the assay kit and presented as *μ*mol/g protein.

### 2.8. Western Blot Analysis

Western blots for the proteins of interest were analyzed as described previously [26]. Briefly, BNST tissues were homogenized, while harvested PC12 cells were lysed in a radioimmunoprecipitation assay lysis buffer containing protease inhibitors. The resultant homogenates were centrifuged at 16,000 ×g for 20 min at 4°C. The supernatants were collected, and their total protein concentrations were measured with the bicinchoninic acid assay. The proteins in the supernatants were separated using sodium dodecyl sulfate polyacrylamide gel electrophoresis and subsequently transferred to polyvinylidene difluoride membranes (Millipore, Bedford, MA, USA). The membranes were sequentially incubated with the primary and secondary antibodies. Finally, the protein bands of interest were visualized with enhanced chemiluminescence (Amersham Biosciences, Little Chalfont, UK), and their densities were quantified using ImageJ software (NIH, Bethesda, MD, USA).

### 2.9. qPCR Analysis

Total RNA was extracted from excised BNST tissue using TRIzol reagent (Invitrogen), and the RNA was converted into cDNA with a reverse transcription PCR kit (Promega, Madison WI, USA). qPCR analysis was carried out using a LightCycler® DNA Master SYBR Green-I kit (Roche Diagnostics, Mannheim, Germany) with a LightCycler 2.0 (Roche Diagnostics). The primers for PCR amplification of iNOS, nNOS, and *β*-actin were as follows: 5′-CAGCTGGGCTGTACAAACCTT-3′ (sense) and 5′-CATTGGAAGTGAAGCGTTTCG-3′ (antisense) for iNOS; 5′-ACCCAACGT CATTTCTGTCC-3′ (sense) and 5′-AAGGTGGTCTCCAGGTGTGT-3′ (antisense) for nNOS; and 5′-GTCGTACCACTGGCATTGTG-3′ (sense) and 5′-GCCATCTCTTGCTCGAAGTC-3′ (antisense) for *β*-actin. The results were normalized to *β*-actin, and relative gene expression was calculated using the 2^−ΔΔCT^ method with the following formula:

(3) $$\begin{array}{c} \Delta \text{CT}={\text{CT}}_{\text{NOS}}-{\text{CT}}_{\beta -\text{actin}}\text{,}\\ \Delta \Delta \text{CT}=\Delta {\text{CT}}_{\text{treated}}-\Delta {\text{CT}}_{\text{vehicle}}. \end{array}$$

### 2.10. Intra-BNST Microinfusion

To determine whether the effects of Sau on EtOHW-induced anxiety were mediated by the BNST NO pathway, SNP, an NO donor, was bilaterally microinfused into the BNST (0.1 nmol, 200 nL per side) 30 min after the third Sau administration. Five minutes later, the rats were tested in the EPM. The SNP was dissolved in modified Ringer's solution (MRS) containing 150 mM NaCl, 3.0 mM KCl, 1.4 mM CaCl_2_, and 0.8 mM MgCl_2_ in 10 mM phosphate buffer (pH = 7.2).

A cohort of male SD rats (280–300 g) was used in this experiment. Anesthetized rats (50 mg/kg sodium pentobarbital, i.p.) were placed onto a stereotaxic frame, and stainless-steel guide cannulas (22-gauge) were bilaterally implanted with their tips 1.5 mm above the BNST. After implantation, the rats were individually housed and allowed to recover for at least 7 days, during which time they were administered antibiotics and analgesics to prevent infection and pain, respectively. Following recovery, the rats were subjected to the same EtOHW (or saline) and drug treatment regimen described above (Figure 1(a)).

Microinfusions were carried out by inserting a 30-gauge injector into each guide cannula; the injectors were 1.5 mm longer than the guide cannulas. SNP was infused over 60 s with a motorized syringe pump. At the end of the experiment, the injection positions of each rat were histologically verified (Figure 1(b)). The treatment groups for this experiment were as follows: saline/vehicle/MRS (*n* = 6), EtOH/vehicle/MRS (*n* = 6), EtOH/Sau25/MRS (*n* = 6), and EtOH/Sau25/SNP (*n* = 6).

### 2.11. Statistical Analysis

Data are presented as the mean ± standard error of the mean (SEM) and were checked for the normality and the homogeneity of variances before further statistical analyses. The data were analyzed using one-way analysis of variance (ANOVA) followed by the Newman–Keuls multiple comparison test, except for the BNST iNOS mRNA levels that were analyzed using a one-tailed unpaired *t*-test. All statistical analyses were performed using GraphPad Prism 5.0 software (GraphPad Software Inc., San Diego, CA, USA). Significant differences were considered when *p* values were <0.05.

## 3. Results

### 3.1. Effects of Sau on Repeated EtOH-Induced Locomotor Sensitization

In previous studies performed by our research team and other researchers, the dosages of Sau used in mice ranged from 2.5 to 30 mg/kg [30, 32, 33]. The dose conversion ratio between mice and rats is approximately 1.43 : 1, and in a preliminary study, we found an acute 30 mg/kg dose of Sau produced small but evident behavioral changes in rats, such as small increases in grooming and gnawing; therefore, in the present study, doses of Sau at 2.5, 7.5, and 25 mg/kg/day were used.

**Figure 2:**
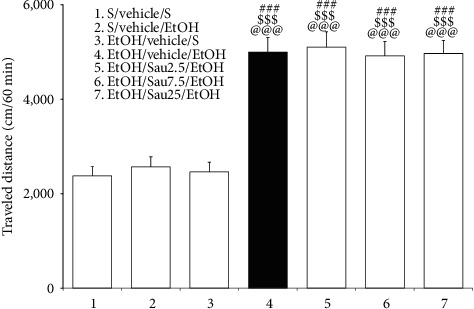
Effects of Sau on repeated EtOH-induced locomotor sensitization. An EtOH challenge 3 days after the cessation of repeated EtOH administrations elicited locomotor sensitization, which was unaffected by Sau treatment during EtOHW. All data are presented as means ± SEM (*n* = 8). S, saline; EtOH, ethanol; Sau, sauchinone; Sau 2.5 : 2.5 mg/kg/day Sau; Sau 7.5, 7.5 mg/kg/day Sau; Sau25, 25 mg/kg/day Sau. ###*p* < 0.001 versus the S/vehicle/S group; $$$*p* < 0.001 versus the S/vehicle/EtOH group; @@@*p* < 0.001 versus the EtOH/vehicle/S group (one-way ANOVA followed by the Newman–Keuls post hoc test).

As shown in Figure 2, on the third day following EtOH treatment cessation, a 0.75 g/kg EtOH challenge significantly increased locomotor activity in EtOH-pretreated rats compared to saline-pretreated rats (*F*_6, 49_ = 25.43, *p* < 0.001; saline/vehicle/saline versus EtOH/vehicle/EtOH, *p* < 0.001; saline/vehicle/EtOH versus EtOH/vehicle/EtOH, *p* < 0.001) and rats challenged with saline (EtOH/vehicle/saline versus EtOH/vehicle/EtOH, *p* < 0.001). The challenge dose of EtOH alone did not significantly increase locomotor activity (saline/vehicle/saline versus saline/vehicle/EtOH, *p* > 0.05). These data indicate that EtOH locomotor sensitization was induced during EtOHW. However, unlike what was expected, post hoc comparison tests revealed that none of the doses of Sau (2.5, 7.5, or 25 mg/kg/day) given during the EtOHW period blocked EtOH locomotor sensitization (EtOH/vehicle/EtOH versus EtOH/Sau2.5/EtOH, EtOH/vehicle/EtOH versus EtOH/Sau7.5/EtOH, EtOH/vehicle/EtOH versus EtOH/Sau25/EtOH, all *p* > 0.05; Figure 2). Additionally, an acute injection of 2.5, 7.5, or 25 mg/kg Sau alone did not significantly alter locomotor activity (data not shown).

### 3.2. Effects of Sau on EtOH Withdrawal-Induced Anxiety-Like Behavior

**Figure 3:**
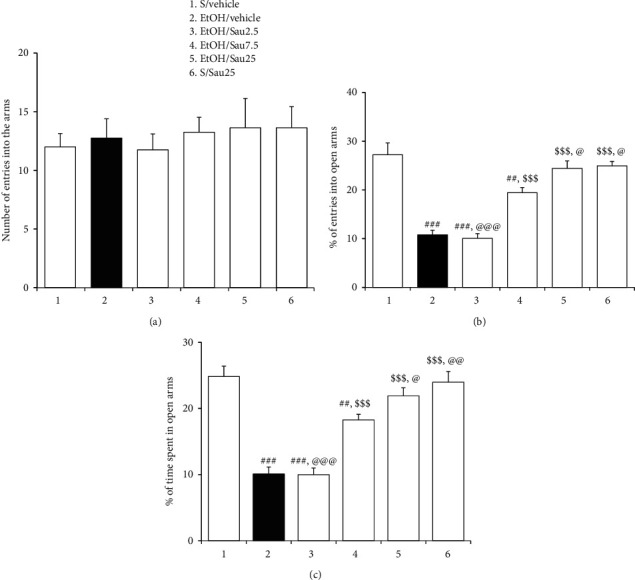
Effects of Sau on EtOH withdrawal-induced anxiety-like behavior. EtOHW produced anxiety-like behaviors in rats when tested 3 days after the final dose of EtOH, but these behaviors were attenuated by Sau treatment during withdrawal. (a) Total number of entries into open and closed arms of the EPM by rats. (b) Percentage of numbers of entries into open arms of the EPM by rats. (c) Percentage of time spent in open arms by rats. All data are presented as a mean ± SEM (*n* = 8). S, saline; EtOH, ethanol; Sau, sauchinone; Sau 2.5, 2.5 mg/kg/day Sau; Sau 7.5: 7.5 mg/kg/day Sau; Sau 25, 25 mg/kg/day Sau. ##*p* < 0.01 and ###*p* < 0.001 versus the S/vehicle group; $$$*p* < 0.001 versus the EtOH/vehicle group; @*p* < 0.05, @@*p* < 0.01, and @@@*p* < 0.001 versus the EtOH/Sau7.5 group (one-way ANOVA followed by the Newman–Keuls post hoc test).

As depicted in Figure 3, EtOHW rats displayed anxiety-like behavior in the EPM when tested 3 days after the final dose of EtOH. Namely, EtOHW rats entered the open arms less frequently and spent less time in them than saline-treated control rats (%entry_open arms_: *F*_5, 42_ = 27.87, *p* < 0.001; saline/vehicle versus EtOH/vehicle, *p* < 0.001; %time_open arms_: *F*_5, 42_ = 28.61, *p* < 0.001; saline/vehicle versus EtOH/vehicle, *p* < 0.001). However, Sau at 7.5 and 25 (but not 2.5) mg/kg/day reversed these anxiety-like behaviors (%entry_open arms_: EtOH/vehicle versus EtOH/Sau7.5, *p* < 0.001; EtOH/vehicle versus EtOH/Sau25, *p* < 0.001; EtOH/vehicle versus EtOH/Sau2.5, *p* > 0.05; %time_open arms_: EtOH/vehicle versus EtOH/Sau7.5, *p* < 0.001; EtOH/vehicle versus EtOH/Sau25, *p* < 0.001; EtOH/vehicle versus EtOH/Sau2.5, *p* > 0.05), and the effects were dose-dependent (%entry_open arms_: EtOH/Sau7.5 versus EtOH/Sau25, *p* < 0.05; %time_open arms_: EtOH/Sau7.5 versus EtOH/Sau25, *p* < 0.05). Additionally, 25 mg/kg/day of Sau alone did not affect anxiety-like behaviors in the EPM (%entry_open arms_: saline/vehicle versus saline/Sau25, *p* > 0.05; %time_open arms_: saline/vehicle versus saline/Sau25, *p* > 0.05; Figure 3).

### 3.3. Effects of Sau on Plasma CORT Levels during EtOHW

**Figure 4:**
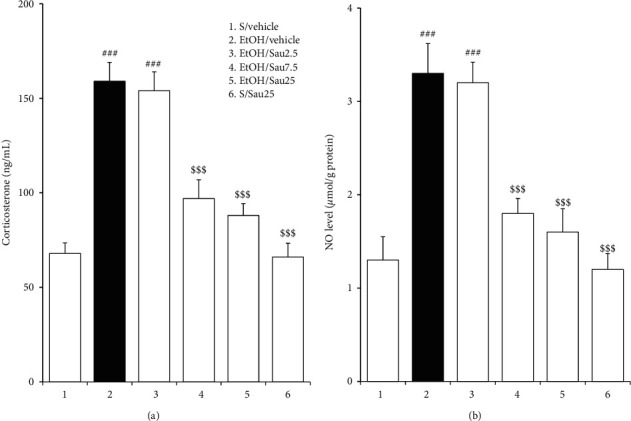
Effects of Sau on plasma CORT levels and NO production in the BNST during EtOHW. Withdrawal from repeated EtOH administration elevated plasma CORT concentrations and NO levels in the BNST in rats, which were both prevented by Sau treatment. All data are presented as means ± SEM (*n* = 7 for CORT, *n* = 6 for NO). S, saline; EtOH, ethanol; Sau, sauchinone; Sau 2.5, 2.5 mg/kg/day Sau; Sau 7.5, 7.5 mg/kg/day Sau; Sau 25, 25 mg/kg/day Sau. ###*p* < 0.001 versus the S/vehicle group; $$$*p* < 0.001 versus the EtOH/vehicle group (one-way ANOVA followed by the Newman–Keuls post hoc test).

Plasma levels of CORT, a hormone indicative of anxiety in rats, increase during EtOHW when challenged by stressors. As shown in Figure 4, plasma CORT levels were significantly increased in EtOH-treated control rats relative to saline-treated controls (*F*_5, 36_ = 24.00, *p* < 0.001; saline/vehicle (*n* = 7) versus EtOH/vehicle (*n* = 7), *p* < 0.001), indicating a state of anxiety in rats during EtOHW. However, the increase was attenuated by Sau treatment at doses of 7.5 and 25 (but not 2.5) mg/kg/day (EtOH/vehicle versus EtOH/Sau7.5 (*n* = 7), *p* < 0.001; EtOH/vehicle versus EtOH/Sau25 (*n* = 7), *p* < 0.001; EtOH/vehicle versus EtOH/Sau2.5 (*n* = 7), *p* > 0.05), analogous to its effects in the EPM. Sau alone at 25 mg/kg/day did not influence plasma CORT levels (saline/vehicle versus saline/Sau25 (*n* = 7), *p* > 0.05; Figure 4(a)).

### 3.4. Effects of Sau on NO Levels in the BNST during EtOHW

As seen in Figure 4(b), 3 days after the final EtOH dose, NO production in the BNST was significantly increased in EtOH-treated control rats compared to their saline-treated counterparts (*F*_5, 30_ = 16.20, *p* < 0.001; saline/vehicle (*n* = 6) versus EtOH/vehicle (*n* = 6), *p* < 0.001), implying elevated NO signaling in the BNST during EtOHW. However, this enhanced signaling was blocked by treatment with Sau at 7.5 or 25 mg/kg/day (EtOH/vehicle versus EtOH/Sau7.5 (*n* = 6), *p* < 0.001; EtOH/vehicle versus EtOH/Sau25 (*n* = 6), *p* < 0.001; EtOH/vehicle versus EtOH/Sau2.5 (*n* = 6), *p* > 0.05). Treatment with Sau alone at 25 mg/kg/day did not significantly affect BNST NO production (saline/vehicle versus saline/Sau25 (*n* = 6), *p* > 0.05; Figure 4(b)).

### 3.5. Effect of Sau on the Protein Expression of CRF, iNOS, and nNOS in the BNST during EtOHW

**Figure 5:**
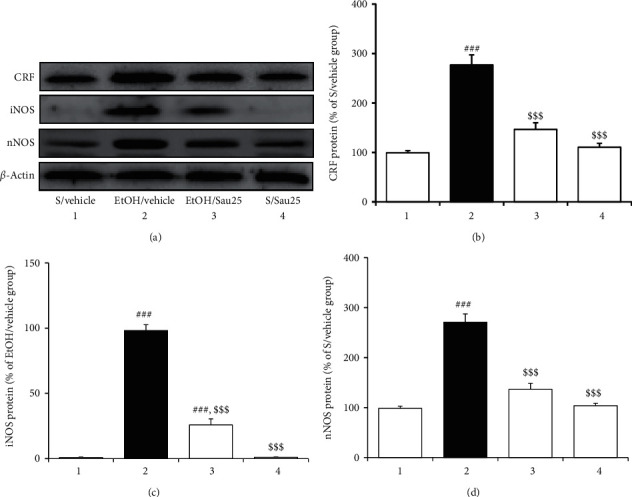
(a) Effect of Sau on the protein expression of CRF, iNOS, and nNOS in the BNST during EtOHW. Immediately after the behavioral test in the EPM, Western blotting analysis was conducted to detect the interested proteins. (b) CRF, (c) iNOS, and (d) nNOS; all data are presented as a mean ± SEM (*n* = 5). S, saline; EtOH, ethanol; Sau, sauchinone; Sau 25, 25 mg/kg/day Sau. ###*p* < 0.001 versus the S/vehicle group; $$$*p* < 0.001 versus the EtOH/vehicle group (one-way ANOVA followed by the Newman–Keuls post hoc test).

CRF protein levels in the BNST have previously been reported to be positively correlated with plasma CORT secretion and anxiety in rats. In this study, as depicted in Figure 5, Western blot analysis showed that EtOHW enhanced CRF protein levels in the BNST (*F*_3, 16_ = 38.96, *p* < 0.001; saline/vehicle (*n* = 5) versus EtOH/vehicle (*n* = 5), *p* < 0.001). However, this enhancement was blocked with Sau treatment at 25 mg/kg/day (EtOH/vehicle versus EtOH/Sau25 (*n* = 5), *p* < 0.001). CRF protein expression in the BNST was not affected by Sau treatment alone at 25 mg/kg/day (saline/vehicle versus saline/Sau25 (*n* = 5), *p* > 0.05; Figure 5).

Western blot analysis revealed that the bands representing the iNOS protein in the saline-treated control and saline/Sau25 groups were barely detectible (Figure 5), likely because the expression of iNOS is induced by inflammatory and immune responses. Nonetheless, protein levels of both iNOS and nNOS in the BNST were significantly increased on the third day of EtOHW (iNOS : *F*_3, 16_ = 205.00, *p* < 0.001; saline/vehicle (*n* = 5) versus EtOH/vehicle (*n* = 5), *p* < 0.001; nNOS : *F*_3, 16_ = 56.97, *p* < 0.001; saline/vehicle (*n* = 5) versus EtOH/vehicle (*n* = 5), *p* < 0.001). The increased iNOS and nNOS expression was reversed by treatment with Sau at 25 mg/kg/day (iNOS: EtOH/vehicle versus EtOH/Sau25 (*n* = 5), *p* < 0.001; nNOS: EtOH/vehicle versus EtOH/Sau25 (*n* = 5), *p* < 0.001). Finally, Sau (25 mg/kg/day) treatment alone affected neither iNOS nor nNOS protein expression in the BNST (iNOS, saline/vehicle versus saline/Sau25 (*n* = 5), *p* > 0.05; nNOS, saline/vehicle versus saline/Sau25 (*n* = 5), *p* > 0.05; Figure 5).

### 3.6. Effect of Sau on the mRNA Expression of iNOS and nNOS in the BNST during EtOHW

**Figure 6:**
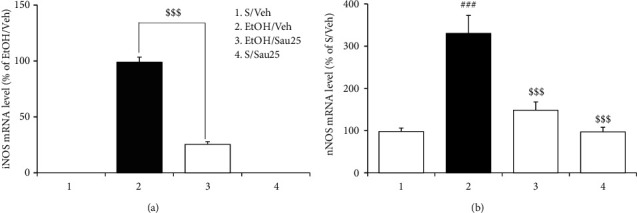
Effect of Sau on the mRNA expression of iNOS and nNOS in the BNST during EtOHW. Immediately after the behavioral test in the EPM, qPCR analysis was performed to measure the mRNA levels of iNOS and nNOS in the BNST. (a) iNOS and (b) nNOS; all data are presented as a mean ± SEM (*n* = 6). S, saline; Veh, vehicle; EtOH, ethanol; Sau, sauchinone; Sau 25, 25 mg/kg/day Sau. ###*p* < 0.001 versus the S/vehicle group; $$$*p* < 0.001 versus the EtOH/vehicle group (a one-tailed unpaired *t*-test for iNOS mRNA; one-way ANOVA followed by the Newman–Keuls post hoc test for nNOS mRNA).

Similar to the abovementioned Western blot analysis, iNOS mRNA expression in the BNST was not observed in either the saline/vehicle or saline/Sau25 group (Figure 6). However, qPCR assays showed that EtOHW induced and elevated the mRNA expression of iNOS and nNOS in the BNST, effects that were significantly inhibited by treatment with Sau (25 mg/kg/day) during EtOHW (iNOS: t_10_ = 14.25, EtOH/vehicle (*n* = 6) versus EtOH/Sau25 (*n* = 6), *p* < 0.001; nNOS: *F*_3, 20_ = 20.65, *p* < 0.001; saline/vehicle (*n* = 6) versus EtOH/vehicle (*n* = 6), *p* < 0.001; EtOH/vehicle versus EtOH/Sau25 (*n* = 6), *p* < 0.001). Sau (25 mg/kg/day) treatment alone did not affect nNOS mRNA expression in the BNST (nNOS: saline/vehicle versus saline/Sau25 (*n* = 6), *p* > 0.05; Figure 6).

### 3.7. Effect of Sau on the Protein Expression of nNOS Induced by H_2_O_2_ in PC12 Cells

Previous studies have reported that the cell viability of PC12 cells significantly decreases when incubated for 24 hours with H_2_O_2_ at doses greater than 100 *μ*M [38, 39], which was also confirmed by our preliminary experiment. Therefore, in this study, the PC12 cells were incubated with 75 *μ*M H_2_O_2_, which did not significantly influence cell viability. The effects of Sau on oxidative stress-induced nNOS expression in these cells were determined with Western blot assays.

**Figure 7:**
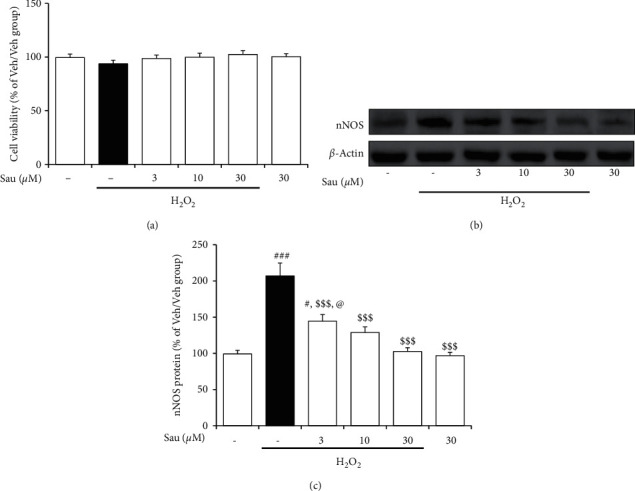
Effect of Sau on the protein expression of nNOS induced by H_2_O_2_ in PC12 cells. PC12 cells were pretreated with 3, 10, and 30 *μ*M of Sau for 60 min and then incubated with 75 *μ*M of H2O2 for 24 h (a) Cell viability was examined with the MTT assay. (b), (c) Western blotting analysis was carried out using PC12 cell lysates. Veh, vehicle; Sau, sauchinone. #*p* < 0.001 and ###*p* < 0.001 versus the Veh/Veh group; $$$*p* < 0.001 versus the Veh/H_2_O_2_ group; @*p* < 0.05 versus the 30 *μ*M Sau/H_2_O_2_ group (one-way ANOVA followed by the Newman–Keuls post hoc test).

Neither 75 *μ*M H_2_O_2_ nor Sau (3, 10, and 30 *μ*M) significantly affected cell viability (*F*_5,__30_ = 0.76, *p* > 0.05; Figure 7). H_2_O_2_ stimulation increased nNOS protein levels in PC12 cells (nNOS: *F*_5, 24_ = 20.08, *p* < 0.001; vehicle/vehicle (*n* = 5) versus vehicle/H_2_O_2_ (*n* = 5), *p* < 0.001; Figure 7). However, similar to the BNST, this increase was attenuated with Sau treatment at 3, 10, or 30 *μ*M (vehicle/H_2_O_2_ versus Sau03/H_2_O_2_ (*n* = 5), *p* < 0.001; vehicle/H_2_O_2_ versus Sau10/H_2_O_2_ (*n* = 5), *p* < 0.001; vehicle/H_2_O_2_ versus Sau30/H_2_O_2_ (*n* = 5), *p* < 0.001) in a dose-dependent manner (Sau03/H_2_O_2_ versus Sau30/H_2_O_2_, *p* < 0.05; Figure 7). Treatment with 30 *μ*M Sau alone did not significantly change nNOS protein expression in the PC12 cells (vehicle/vehicle versus Sau30/vehicle (*n* = 5), *p* > 0.05; Figure 7).

### 3.8. Effect of Intra-BNST Infusions of SNP on the Anxiolytic Action of Sau during EtOHW

**Figure 8:**
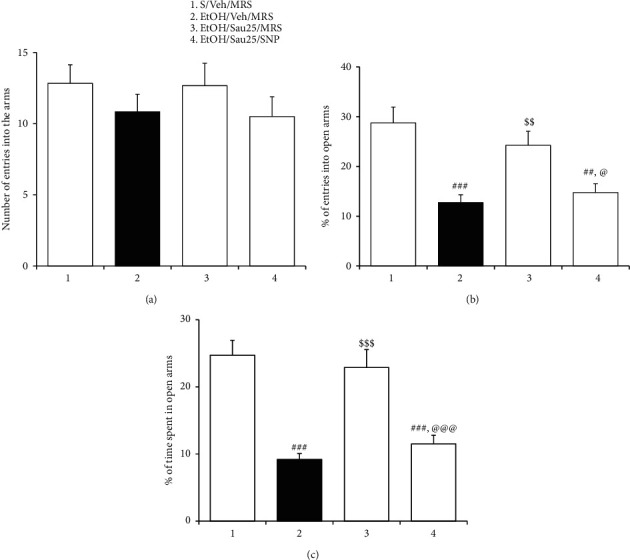
Effect of intra-BNST infusions of SNP on the anxiolytic action of Sau during EtOHW. At 30 min after the third dose of 25 mg/kg/dad Sau, the rats received bilateral intra-BNST infusions of SNP and then were tested in the EPM for evaluating anxiety-like behavior. (a) Total number of entries into open and closed arms of the EPM by rats. (b) Percentage of numbers of entries into open arms of the EPM by rats. (c) Percentage of time spent in open arms by rats. All data are presented as a mean ± SEM (*n* = 6). S, saline; EtOH, ethanol; MRS, modified Ringers' solution; Sau, sauchinone; Sau 25, 25 mg/kg/day Sau. ##*p* < 0.01 and ###*p* < 0.001 versus the S/vehicle/MRS group; $$*p* < 0.01 and $$$*p* < 0.001 versus the EtOH/vehicle/MRS group; @*p* < 0.05 and @@@*p* < 0.001 versus the EtOH/Sau25/MRS group (one-way ANOVA followed by the Newman–Keuls post hoc test).

After intra-BNST infusions of MRS or SNP, the rats were tested in the EPM. EtOH-treated control rats displayed anxiety-like behaviors that were inhibited by treatment with Sau at 25 mg/kg/day (%entry_open arms_: *F*_3, 20_ = 9.90, *p* < 0.001; saline/vehicle/MRS (*n* = 6) versus EtOH/vehicle/MRS (*n* = 6), *p* < 0.001; EtOH/vehicle/MRS versus EtOH/Sau25/MRS (*n* = 6), *p* < 0.01; % time_open arms_: *F*_3, 20_ = 17.02, *p* < 0.001; saline/vehicle/MRS versus EtOH/vehicle/MRS, *p* < 0.001; EtOH/vehicle/MRS versus EtOH/Sau25/MRS, *p* < 0.001; Figure 8), consistent with the behavioral findings described above. However, the anxiolytic-like actions of Sau were abrogated when SNP was injected into the BNST after the third dose of Sau (%entry_open arms_: EtOH/Sau25/MRS versus EtOH/Sau25/SNP (*n* = 6), *p* < 0.05; %time_open arms_: EtOH/Sau25/MRS versus EtOH/Sau25/SNP, *p* < 0.001; Figure 8).

## 4. Discussion

Previous studies have demonstrated that Sau improves behavioral and pathological signs of methamphetamine dependence [32, 33]; accordingly, in this study, the effects of Sau on EtOH dependence were evaluated using rat EtOH locomotor sensitization and withdrawal anxiety models.

When rats were treated with Sau (2.5, 7.5, and 25 mg/kg/day), daily during a 3-day EtOHW period, Sau at all three doses had no effect on EtOH locomotor sensitization. In contrast, Sau at 7.5 and 25 (but not 2.5) mg/kg/day alleviated EtOHW anxiety in the EPM in a dose-dependent manner. Accordingly, Sau at 7.5 or 25 mg/kg/day blocked the elevation of plasma CORT levels and NO production in the BNST during EtOHW, and Sau at 25 mg/kg/day decreased CRF protein expression in the BNST. In the Western blot assays, Sau attenuated increases in the protein expression of both iNOS and nNOS in the BNST during EtOHW and inhibited H_2_O_2_-stimulated nNOS protein expression in PC12 cells. Correspondingly, Sau reduced the increased mRNA expression of iNOS and nNOS in the BNST during EtOHW. In the local infusion experiment, the injection of SNP into the BNST following Sau administration abolished the anxiolytic action of Sau in EtOHW. Taken together, these results suggest that Sau, when administered during EtOHW, can attenuate EtOHW-induced anxiety without affecting EtOH locomotor sensitization, and its anxiolytic effects are mediated by inhibition of NO signaling in the BNST.

Behavioral sensitization of EtOH has been reported in some rodent strains, such as DBA/2J mice and SD rats [36, 40]. For example, Hoshaw and Lewis reported locomotor sensitization in SD rats challenged by EtOH 21 days after a 15-day period of i.p. EtOH administration [36]. In this study, when challenged by EtOH 3 days after a 28-day EtOH administration protocol, EtOH-pretreated rats traveled greater distances than saline-pretreated rats, indicating EtOH locomotor sensitization. This behavioral sensitization is neurochemically supported by our previous study reporting that SD rats undergoing the same EtOH regime as that used in this study showed sensitized accumbal dopamine release when challenged with EtOH [4]. Moreover, it was previously demonstrated that Sau at 5 or 10 mg/kg attenuated methamphetamine-induced hyperlocomotion, with the latter dose also blocking methamphetamine-conditioned place preference [32]. However, in this study, none of the three doses (2.5, 7.5, and 25 mg/kg/day) of Sau significantly influenced EtOH locomotor sensitization. The development of EtOH locomotor sensitization is divided into three phases: the acquisition phase (daily EtOH intake), the incubation phase (the withdrawal period), and the expression phase (immediately after EtOH challenge). Among these, the incubation phase mimics the increasing intensity of EtOH cravings after abstinence; hence, Sau was administered to rats during EtOHW in this study. The results from this study indicated that Sau treatment during EtOHW cannot block the development of EtOH locomotor sensitization, that is, it cannot block the positive reinforcing effects of EtOH during abstinence. These results also implied that the mechanisms underlying positive reinforcement-related behavioral changes likely differ between EtOH and methamphetamine.

Rats show increased anxiety-like behaviors in the EPM during a certain period of EtOHW [41]. The EtOHW paradigm used in this study has been well validated [12, 42]. Accordingly, substantial anxiety-like behaviors in the EPM in EtOHW rats were observed. EtOHW rats visited the open arms less frequently and spent less time in them compared to saline-treated control rats. These anxiety-like behaviors were dose-dependently reversed with Sau treatment at 7.5 or 25 mg/kg/day, that is, Sau increased the number of entries made and the duration of time spent in the open arms, by EtOHW rats. These results indicated that Sau administered during EtOHW can attenuate withdrawal-induced anxiety. Although Sau itself has not yet been reported to exert anxiolytic actions, lignans isolated from *Schisandra chinensis* fruit have been demonstrated to reduce restraint stress-induced anxiety [43]. Furthermore, in mice, Sau has been shown to rescue repeated methamphetamine-induced damage to striatal dopaminergic terminals [33], implying that it may alleviate the emotional disturbances induced by drug abuse. In this study, the anxiolytic effects of Sau during EtOHW were further corroborated by the finding that Sau treatment blocked EtOHW-induced increases in the plasma CORT concentration and CRF protein level in the BNST; oversecretion of CORT and CRF in blood and the BNST, respectively, are the hormonal and neurochemical hallmarks of anxiety in rats [12, 44]. Taken together, these results indicate that Sau inhibits EtOHW-induced anxiety.

The BNST is a key brain structure in the anxiety-like behaviors induced in rodents by drugs of abuse, and these anxiety-like behaviors are mediated by enhanced CRF signaling in the BNST [44, 45]. The BNST is innervated by a variety of neurotransmitter, neuromodulator, and neuropeptide systems that modulate CRF signaling and therefore affect the manifestation of anxiety states [46, 47]. Inhibition of neural NO production has anxiolytic effects, and the neural NO system modulates central CRF signaling [23, 48]. Indeed, Faria et al. reported that increased NO production in the BNST induced anxiety that was mediated by the CRF/CRF1R (CRF1 receptor) signaling pathway [20]. In the present study, EtOHW promoted NO production in the BNST; this effect was reversed by Sau treatment (7.5 or 25 mg/kg/day), similar to how Sau inhibited CRF protein expression in the BNST. A previous study reported that Sau reduced lipopolysaccharide-stimulated NO production in microglia [35]. Taken together, these findings indicate that the anxiolytic effects of Sau may be mediated by the NO signaling pathway in the BNST.

NO production is catalyzed by three NOS isoforms: nNOS, endothelial NOS (eNOS), and iNOS. nNOS and eNOS are constitutively expressed, while the expression of iNOS is induced by inflammatory and immune responses. iNOS is mainly responsible for the amount of NO production [49]. In this study, EtOHW increased both the mRNA and protein expression of iNOS in the BNST, indicating enhanced NO production and corroborating the findings of Bonassori et al. who reported that increased neural iNOS activity was associated with EtOHW-induced anxiety [50]. Bonassori et al. also observed that nNOS, but not eNOS, is involved in EtOHW anxiety [51]. Likewise, in this study, EtOHW significantly elevated nNOS gene and protein expression in the BNST, without affecting eNOS expression (data not shown). However, treatment with 25 mg/kg/day Sau did attenuate EtOHW-induced increases in the mRNA and protein expression of both iNOS and nNOS.

The inhibitory effect of Sau on iNOS has been well documented in in vitro experiments using Raw264.7 (a murine macrophage cell line) and BV2 cells (a murine microglial cell line) [29, 35]; however, its action on nNOS function was previously unknown. Hence, this study was the first to report that Sau inhibited nNOS expression in vivo. To further elucidate this effect and because excessive oxidative stress during EtOHW contributes to affective disorders [52], neuronal PC12 cells were used, with H_2_O_2_ acting as an oxidative stressor. H_2_O_2_ stimulation increased nNOS protein expression in PC12 cells [38, 39], which was dose-dependently inhibited by Sau at doses of 3, 10, and 30 *μ*M in the present study. These results indicated that Sau may inhibit the expression of iNOS and nNOS in the BNST during EtOHW to reduce NO production, thereby mediating its anxiolytic effects.

Finally, the role of NO in the BNST on the anxiolytic effect of Sau during EtOHW was pharmacologically tested. Infusions of SNP into the BNST after Sau administration blocked the anxiolytic effects of Sau in the EPM. This observation, together with the results from the abovementioned biochemical assays, indicated that Sau attenuates EtOHW anxiety by inhibiting NOS-NO-CRF signaling in the BNST.

In summary, this study found that Sau treatment during withdrawal mitigated EtOHW anxiety, but did not influence EtOH locomotor sensitization. Moreover, the anxiolytic effects of Sau were mediated by modulation of NO signaling in the BNST. These findings provide experimental evidence that Sau can block the negative reinforcing effects of EtOH and therefore may be a candidate for alcoholism treatment. Given that the dependence induced by some drugs of abuse, such as nicotine and morphine, also involves central NO, and future research on the effects of Sau on these drugs may be fruitful.

## Data Availability

The data that support the findings of this study are statistically analyzed and included within the article and are available from the corresponding author upon request.
